# Age-related changes in crowding and reading speed

**DOI:** 10.1038/s41598-017-08652-0

**Published:** 2017-08-15

**Authors:** Rong Liu, Bhavika N. Patel, MiYoung Kwon

**Affiliations:** Department of Ophthalmology, School of Medicine, University of Alabama at Birmingham, Birmingham, AL USA

## Abstract

Crowding, the inability to recognize objects in clutter, is known to play a role in developmental changes in reading speed. Here, we investigated whether crowding also plays a role in age-related changes in reading speed. We recruited 18 young (mean age: 22.6 ± 3.5; range: 18~31) and 21 older adults (mean age: 58.2 ± 7.0; range: 50~73) with normal vision. Reading speed was measured with short blocks of text. The degree of crowding was determined by measuring crowding zone (the distance between a target and flankers required to yield a criterion recognition accuracy) and the size of the visual span (an uncrowded window in the visual field within which letters can be recognizable reliably). Measurements were made across the central 16-degree visual field using letter-recognition tasks. Our results showed that, compared to young adults, older adults exhibited significantly slower reading speed (a decrease by 30%) and larger crowding: an enlargement of crowding zone (an increase by 31%) and shrinkage of the visual span (a decrease by 6.25 bits). We also observed significant correlations between reading speed and each of the crowding measures. Our results suggest that crowding increases with age. Age-related changes in crowding may in part explain slower reading in older adults.

## Introduction

Reading is indispensable to many daily activities, affecting a person’s ability to function at work and at home^1^, and is thus a major component of vision-related quality of life^2^. Yet older adults often report difficulty reading^1^. A number of studies have shown age-related decline in reading speed^3, 4^ despite normal vision^5–9^. As society is aging rapidly^10^, having a better understanding of the perceptual mechanisms underlying reading difficulties in the aging population is a necessary step toward developing effective reading rehabilitation for this population. The current study was thus undertaken to identify a potential limiting factor underlying age-related changes in reading speed.

**Figure 1:**
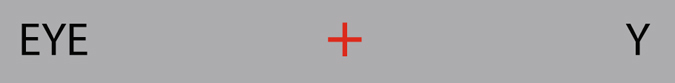
Demonstration of visual crowding. While fixating on the red cross, a person can easily identify the isolated letter on the right, but find it impossible to identify the middle letter on the left. The nearby letters, ‘E’s, disrupt recognition of the target.

Visual crowding, the inability to recognize a target due to the deleterious effect of nearby clutter (See Fig. 1)^11–13^, is known to set a fundamental limit on a variety of visual tasks, such as visual search or object recognition^14–21^. Empirical studies have shown that crowding also plays a significant role in developmental changes in reading speed. For example, a study done by Kwon *et al*.^22^ showed that developmental increases in reading speed in English-speaking school-age children were largely accounted for by a reduction in crowding. Similar results were also found in the reading development of French-speaking children^23^. However, the role of crowding in age-related changes in reading remains unknown.

As demonstrated in Fig. 1, the observer has no trouble recognizing the single letter presented alone (“Y” on the right), but experiences a great deal of difficulty identifying the middle letter (“Y” on the left) flanked by ‘E’s despite the same eccentricity. Hence, crowding cannot be simply explained by reduced acuity or loss of contrast sensitivity. Among many accounts of crowding^24–30^, a popular explanation is that features of the target and flankers are integrated inappropriately due to a larger integration zone (or perceptive pooling zone)^20, 31–39^, which results in relevant features being perceptually indistinguishable. Consistent with this view, crowding grows with increasing retinal eccentricity as receptive field size increases in the periphery (i.e., scale shift)^11, 40, 41^. On the other hand, it has been proposed that loss of retinal ganglion cells may lead to enlargement of receptive field size^42^, suggesting possible changes in cortical pooling mechanisms^43^. Thus, considering the fact that normal aging is associated with gradual loss of retinal ganglion cells and their axons^44–47^, it is not unlikely that age-related loss of ganglion cells exacerbates the crowding effect in older adults. In fact, brain imaging studies showed that the size of population receptive fields in human early visual cortex increases with aging^48, 49^. Despite conflicting evidence^50, 51^, a number of behavioral studies have shown that the age difference in simple visual search or recognition tasks, or even in perceived target contrast, becomes more evident when targets are presented amid nearby distractors^52–60^ or surrounds^61, 62^. Taken together, the findings of these studies suggested the susceptibility of older adults to crowded visual environments.

While these findings together hint at a possible role of crowding in age-related decline in reading, this question still remains to be answered. Thus, the purpose of the current study was to see if crowding increases with age and to see if an increase in crowding (if any) covaries with age-related decline in reading speed. To this end, crowding and reading speed were measured in two age groups–young and older adults. The crowding effect was assessed with two well-established methods: i) the spatial extent of crowding (i.e., crowding zone)^11, 13, 40, 63^, which refers to the minimum spacing between a target and flankers within which target identification remains unaffected by the presence of distractors. Thus, the greater the influence of crowding, the larger the crowding zone. As crowding zone approximates the extent of spatial interference of distractors, it has been commonly used to measure crowding^11, 21, 25, 40^; and ii) the size of the visual span (i.e., the number of letters that can be reliably recognizable in a glance)^64, 65^. Because the size of the visual span is largely accounted for by crowding^21, 66–69^, the visual span is also called the “uncrowded window”^70^ in visual space. Furthermore, as the size of the visual span has been shown to be a good predictor of reading speed in both normal and clinical populations^64, 67, 71–73^, the visual span measure is a natural choice for studying the role of crowding in reading speed. In the current study, crowding measurements were made at multiple retinal locations, spanning 16-degree central visual field. We then compared differences in reading speed, crowding zone, and the size of the visual span between the two age groups and also examined the relationship between crowding (i.e., crowding zone and the visual span) and reading speed.

## Results

### Slower reading speed in older adults

**Figure 2:**
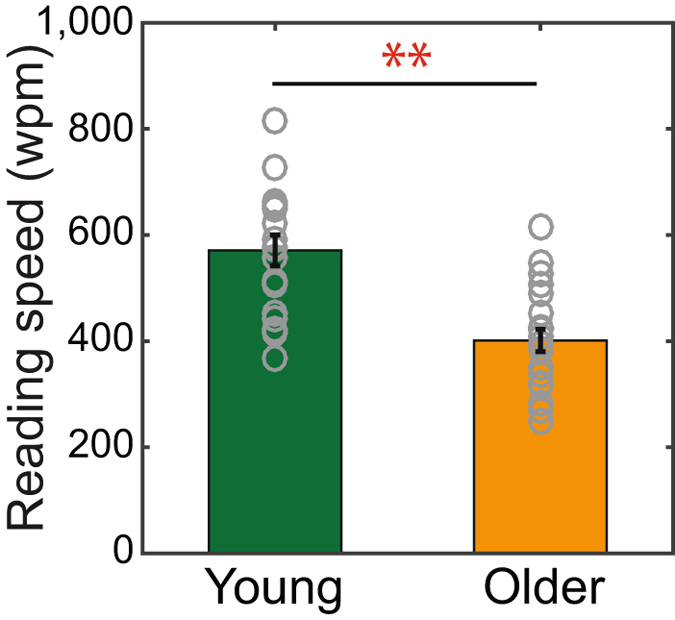
Slower reading speed in older adults compared to young adults. Mean reading speed (words per minute, wpm) was plotted as a function of age group. The green bar represents the mean reading speed of the young adults while the orange bar indicates the mean reading speed of the older adults. Each gray open circle represents the date point from each individual subject. ‘**’ denotes *p* < 0.01. Error bars represent ± 1 standard error of the mean (SEM).

Figure 2 plots the mean reading speed (in words per minute, wpm) as a function of age group. Consistent with previous findings^3–9^, we observed that older adults (orange bar) read significantly slower than young adults (green bar) (mean ± standard error: 570.95 ± 29.03 vs. 401.30 ± 21.03 wpm, *t*_(37)_ = 4.82, *p* < 0.01).

### Enlarged crowding zone in older adults

**Figure 3:**
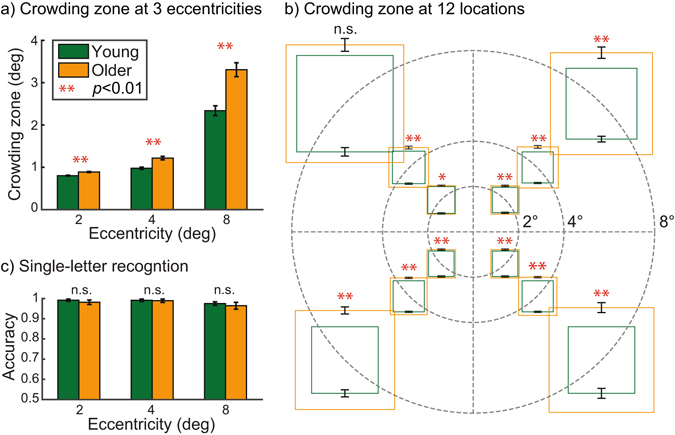
Enlarged crowding zone in older adults compared to young adults. (**a**) Mean crowding zone for three eccentricities. Green bars indicate the data from the young adults and orange bars indicate the one from the older adults. (**b**) Mean crowding zone plotted in the polar coordinate plane for the 12 testing locations. Each dotted circle represents each eccentricity (2°, 4°, or 8°). Green squares represent the crowding zones for the young adults while orange squares represent those for the older adults. (**c**) Mean single-letter recognition accuracy for three eccentricities. ‘n.s.’ denotes no statistical significance. ‘*’ denotes *p* < 0.05. ‘**’ denotes *p* < 0.01. Error bars represent ±1 SEM.

As shown in Fig. 3a, older adults exhibited significantly larger crowding zone compared to young adults across all eccentricities (*F*_(1,37)_ = 24.81, *p* < 0.01, an increase by 31% on average). Consistent with earlier findings^11, 40^, crowding zone increased with increasing eccentricity for both age groups (*F*_(2,74)_ = 398.98, *p* < 0.01). In Fig. 3b, the crowding zone of each testing location was plotted in the polar coordinate plane. The difference in crowding zone between the two groups remained significant across all testing locations (*p*s < 0.05) except for the location in the upper left quadrant at 8° eccentricity. It is also noteworthy that there was no significant difference in single-letter recognition performance between the two groups (99% vs. 98% recognition accuracy, *F*_(1,37)_ = 0.57, *p* = 0.45) as summarized in Fig. 3c. These results helped us rule out age-related decline in either visual acuity, contrast sensitivity, or task-specific attention as potential explanations of the increased crowding in older adults because older adults had no trouble recognizing the same target letter ( >96% recognition accuracy) when it was presented alone.

### Shrinkage of the visual span in older adults

**Figure 4:**
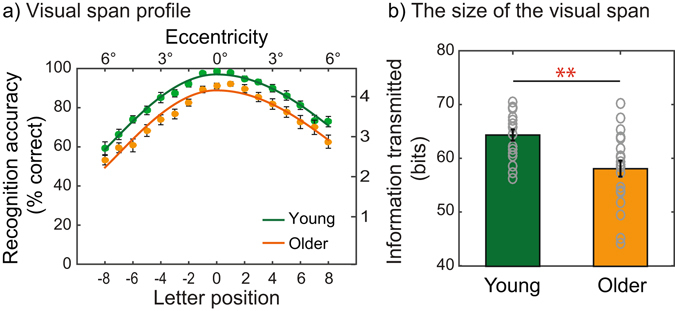
Shrinkage of the visual span in older adults compared to young adults. (**a**) Mean visual span profiles. Letter recognition accuracy was plotted as a function of letter position for the young adults (green dots) and the older adults (orange dots). The solid (green or orange) line represents a split Gaussian function fitted to the average visual profile for the young or older group, respectively. (**b**) Mean visual span size for the two age groups. Each gray open circle represents the date point from each individual subject. ‘**’ denotes *p* < 0.01. Error bars represent ±1 SEM.

Figure 4 plots the mean visual span profiles (Fig. 4a) and the mean size of the visual span (Fig. 4b) for the young and older adults. The green and orange symbols indicate young adults and older adults, respectively. As a reminder, the visual span profile was measured using a trigram-letter recognition task in which subjects recognized trigrams (i.e., random strings of three letters) presented at different letter positions. As expected, accuracy of letter recognition fell off with increasing eccentricity (Fig. 4a) for both age groups. Importantly, the data from the older adults showed a noticeably lower visual-span profile, resulting in a significantly smaller visual span compared to that of the young adults (58.17 ± 1.48 vs. 64.42 ± 1.04 bits, *t*_(37)_ = 3.36, *p* < 0.01). Considering the fact that 100% correct recognition of one letter requires 4.7 bits of information^71^, a decrease by 6.25 bits of information in older adults means that older adults tend to recognize at least 1.3 letters less than what young adults would recognize at one glance.

### Relationship between crowding and reading speed

**Figure 5:**
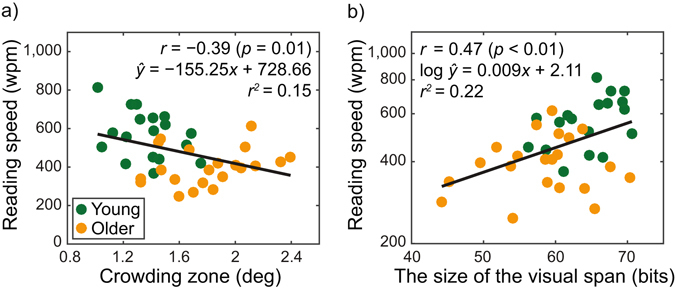
Relationship between reading speed and crowding. (**a)** Correlation between reading speed (wpm) and crowding zone (deg). (**b)** Correlation between log reading speed (wpm) and the size of the visual span (bits). The green and orange symbols indicate young adults and older adults, respectively. The black lines represent the best linear fits to the corresponding data.

Figure 5 shows correlation results between reading speed and crowding. Note that the logarithmic (log) reading speed was compared to the size of the visual span because the visual span was reported in a log unit (i.e., bits of information transmitted). Each data point in Fig. 5a represents an average crowding zone data across all 12 testing locations for each individual subject. The green and orange symbols indicate young adults and older adults, respectively. The black solid lines represent the best linear fits to the corresponding data. Reading speed was significantly correlated with crowding zone (*r* = −0.39, *p* = 0.01) and the size of the visual span (*r* = 0.47, *p* < 0.01), respectively. It is also worth noting that, the correlation between reading speed and crowding zone becomes much stronger (*r* = −0.48, *p* < 0.01) when we only consider the crowding zone measured within 2° eccentricity. This result suggests crowding near the fovea likely has a greater impact on reading speed. Furthermore, the regression of reading speed on both crowding zone and the size of the visual span revealed that approximately 15% (*r* ^2^ = 0.15) to 22% (*r* ^2^ = 0.22) of the variance in reading speed could be accounted for by crowding.

We, however, did not find any significant correlation between visual acuity and reading speed (*r* = −0.19, *p* = 0.22), nor between contrast sensitivity and reading speed (*r* = 0.27, *p* = 0.10). Consistent with our single-letter recognition results (Fig. 3c), these correlation results further confirmed that visual crowding, rather than age-related decline in visual acuity and contrast sensitivity, was likely to account for the observed difference in reading speed between the two age groups.

## Discussion

Despite conflicting evidence^50, 51^, many studies^52–62^ have shown that older adults tend to be more vulnerable to the effects of irrelevant visual information such as distracting surrounds. We, thus, hypothesized that age-related decline in reading speed^5–9^ might be related to age-related increase in crowding, as crowding has been shown to play a limiting role in various visual recognition including reading^14–21^. To address this question, we compared reading speed and crowding between two age groups–young and older adults. In the current study, the crowding effect was quantified as crowding zone^11, 13, 25, 40, 63^. In addition, we also measured the visual span because it is known to be highly correlated with reading speed^64, 67, 71–73^ while being largely limited by crowding^21, 66–69^.

First, consistent with previous studies^3–9^, we observed a significant reduction in reading speed in older adults compared to young adults. Our results showed that older adults (mean age 58 years old, range 50–73 years) read significantly slower (by 30%) compared to our young adults (mean age 23 years old, range 18–31 years). Our results, however, showed a much larger decrease in reading speed (a decrease by 30%) in older adults compared to the study of Calabrese *et al*.^9^ (a decrease by approximately 13%). This apparent discrepancy might be due to some obvious methodological differences in measuring reading speed between the two studies. MNREAD charts were used in the study of Calabrese *et al*.^9^, whereas the flashcard method^22, 74, 75^ was used in our study. Unlike the MNREAD charts, in the flashcard reading test, each sentence was displayed on a computer screen only for a fixed exposure duration during which subjects were asked to read as many words as possible (see a more detailed description in Methods). Because of this, the flashcard method often yields a much faster reading speed than the MNREAD charts^9, 22, 74–76^ even for the same subject (e.g., ~200 wpm for MNREAD reading speed vs. ~600 wpm for Flashcard reading speed even for the same group of young subjects in the study of Yu *et al*.^74^. Also see Primativo *et al*.’s^77^ for variability in reading speed estimates across different measurement methods). Thus, it is possible that our flashcard reading measure might have amplified any potential difference in reading speed between older and younger adults. However, we cannot rule out other possible factors, including using a chart-based vs. computer-based test, that might have contributed to the discrepancy.

Second, we observed a significant increase in crowding in older adults – enlargement of crowding zone (an increase by 31%) and shrinkage of the visual span (a decrease by 6 bits). Our findings are consistent with previous studies^51–62^ showing the vulnerability of older adults to the effects of irrelevant visual information such as distracting surrounds. However, a couple of other studies have reported no significant age-related increase in crowding. It is possible that the failure to find an association between age and crowding might be related to the way crowding was measured (e.g., different testing stimuli or locations). For instance, Scialfa *et al*.^57^ showed that orientation discrimination of a Landolt C gap was much worse when the target letter was flanked by vertical bars (crowded condition) in older adults compared to younger adults. On the other hand, studies done by Malavita *et al*.^51^ and Astle *et al*.^50^ reported no significant increase in crowding in older adults. In the study of Malavita *et al*.^51^ crowding was defined as the ratio of the flanked to unflanked spatial-frequency threshold for discriminating the orientation of a Gabor. However, no significant difference was found in crowding ratio between young (18–32 years old) and older adults (60–74 years old). The discrepancy between our results and those of Malavita *et al*.^51^ might be in part due to some noticeable methodological differences between the two studies, including testing stimulus (Gabor patches for Malavita *et al*.’s vs. letters in the English alphabet for ours), task (Gabor orientation discrimination vs. letter identification), operational definition of crowding (crowding ratio vs. crowding zone), and testing location (8° eccentricity in the right/left visual field vs. 12 different retinal locations across the central 16 degrees of visual field). On the other hand, Astle *et al*.^50^ measured crowding zone using a letter recognition task similar to our current method. It is not clear what might have caused discrepant findings between our study and those of Astle *et al*.^50^. However, it should be noted that in Astle *et al*.’s study^50^, crowding zone was measured at only one retinal location in the upper visual field (10° above a fixation point). Interestingly, our results also showed that the differences in crowding zone between the two age groups at one testing location at 8° eccentricity in the upper visual field was not statistically significant (see Fig. 3b). It thus appears that age-related changes in crowding are not constant across the visual field, as crowding itself is spatially inhomogeneous throughout the visual field^41, 63, 78^. Therefore, the question of whether age-related changes in crowding are, to some degree, visual-field dependent calls for a future study.

While the exact mechanism mediating crowding is still a matter of debate^12, 79^, it is often explained by inappropriate integration of features due to a larger integration zone^13, 26, 31, 32, 34, 38, 63, 80–82^. Brain imaging studies indeed showed that the size of population receptive fields in human early visual cortex increase with aging^48, 49^, which may be attributed to age-related loss of retinal ganglion cells^44–47^ or the reduction in the efficacy of inhibitory interactions^83–85^. For example, Curcio and Drucker showed that the density of retinal ganglion cells subserving the central 11 degrees of vision was reduced by one-fourth in healthy older adults compared to younger adults^44^. Perhaps, this loss contributes to alterations in cortical pooling mechanisms in aged individuals. This view is indeed consistent with evidence from glaucoma patients^43, 45, 86^ and animal models of glaucoma^42^, suggesting the inverse relationships between the number of retinal ganglion cells and the size of spatial integration zone. Furthermore, in our recent study^87^, we have shown that the variations in both Ricco’s area (i.e., the area of complete spatial summation) and the spatial extent of crowding across the visual field could be largely explained by the variations in retinal ganglion cell density across the retina. For example, the asymmetry in both Ricco’s area and crowding zone between the upper and lower visual fields were accounted for by the asymmetry in retinal ganglion cell density between the superior and inferior retina. These results suggest a close linkage between retinal ganglion cell density and the size of spatial integration zone (Ricco’s area or crowding zone). These results predicted that the crowding zone likely increases as retinal ganglion cell density decreases. We indeed found a significant reduction (by ~10%, *p* < 0.05) in the thickness of retinal ganglion cell plus inner plexiform (RGC + ) layer in the macular region of the eye in normally-sighted older adults (mean age = 61 yrs) compared to young adults (mean age = 20 yrs). Consistent with earlier studies^44–47^, our results clearly demonstrated a significant age-related decline in retinal ganglion cell density. Thus, it is possible that age-related loss of ganglion cells may play a role in exacerbating the crowding effect in older adults.

Importantly, as shown in Fig. 3c, we did not observe any noticeable difference in single-letter recognition between the two age groups (*p* = 0.45). In other words, older adults had no trouble recognizing the target letter ( >96% recognition accuracy) when it was presented alone (without flankers) regardless of retinal eccentricity. Thus, the increased crowding we observed in older adults did not appear to be due to age-related decline in either visual acuity, contrast sensitivity, or attention to task-relevant stimuli. Our correlation analyses further confirmed that neither visual acuity (*r* = −0.19, *p* = 0.22) nor contrast sensitivity (*r* = 0.27, *p* = 0.10) was likely to account for the observed difference in reading speed between the two age groups. Of course, it does not mean that neither acuity nor contrast sensitivity plays any limiting role in reading speed per se. What our results tell us is that at least for a moderate amount of age-related decline in acuity and contrast sensitivity, visual crowding appears to be the major limiting factor in age-related decline in reading speed. It is also worth noting that both younger and older adults did have fairly good acuity (better than 20/20 on average) and contrast sensitivity ( >1.90 log unit on average).

Third, consistent with previous findings^21, 64, 67, 69, 71, 72, 88–92^, our results also showed that reading speed was significantly correlated with either crowding zone (*r* = −0.39, *p* = 0.01) or the visual span (*r* = 0.47, *p* < 0.01). The relationship between reading speed and visual span was noticeably stronger compared to the relationship between reading speed and crowding zone. This may be in part due to the difference in testing locations between the two measures: the visual span was tested along the horizontal meridian, while crowding zone was measured along the oblique meridian. As English is read from left to right, the crowding effect along the horizontal meridian may exert a stronger influence on reading compared to the crowding effect along the oblique meridian.

In the current study, we measured reading speed using simple, standardized sentences with text difficulty below 3^rd^ grade level. Participants were asked to read aloud instead of silently. This method allows us to address the front-end visual aspects of reading, while minimizing other high-level cognitive and linguistic influences. Reading speed has been proven to be a functionally significant measure, as slow and effortful reading in impaired vision often reflects a bottom-up, visual sensory limitation on reading^68^. While the focus of our study was on the role of visual factors (i.e., crowding zone or the visual span) in age-related decline in reading, it is also possible that other factors such as age-related decline in oculomotor control^3, 6, 93–95^ might have been contributed to slower reading speed in older adults. As shown in our results, visual crowding explained approximately 15% to 22% of the variance in our Flashcard reading speed. Although it is not likely (for the reasons mentioned above), it is still possible that non-visual factors such as education or reading experience may in part explain the remaining unexplained variance in reading speed.

We acknowledge that future studies should consider including a wider range of age groups (e.g., individuals aged between 30 and 50 years old, and individuals aged 75 or older) in its design, which allows for detailed characterization of the dependency of crowding and reading speed on age and the correlation between the two. Future studies should also consider acquiring additional information on reading experience (e.g., the amount time spent on reading per day) and education in order to control for any potential confounders. While the current study used the Flashcard method to assess reading speed, it is also important to verify whether the pattern of results found in the current study can be generalized to other types of reading speed measures (e.g., normal silent reading). Despite these limitations, our study is the first attempt to demonstrate a potential role of crowding in age-related decline in reading. Our findings also have implications for understanding the perceptual mechanisms underlying age-related deficits in everyday visual function, such as driving and visual search.

In summary, our results showed that older adults exhibit an enlargement in crowding zone and shrinkage of the visual span compared to their young counterparts. Our findings suggest that age-related decline in the ability to recognize targets in clutter may in part explain slower reading speed in older adults.

## Methods

### Participants

Study participants included 18 young (age range 18–31 years, mean 22.6 ± 3.5 years, mean ± standard deviation, 6 males) and 21 older adults (age range 50–73 years, mean 58.2 ± 7.0 years, 12 males). All participants were recruited from the Birmingham metropolitan area. They were native English speakers without known cognitive or neurological impairments. They had normal or corrected to normal vision. Normal vision was defined as having better than or equal to 0.1 logMAR (equivalent to 20/25) corrected visual acuity, normal contrast sensitivity, normal stereoacuity, and no known visual disorder. Proper refractive correction for the viewing distance was used. Mean acuity (ETDRS Chart) was −0.16 ± 0.08 logMAR for young adults and −0.09 ± 0.07 logMAR for older adults. Mean contrast sensitivity (Pelli-Robson Contrast Sensitivity Chart) was 1.97 ± 0.08 log units for young adults and 1.93 ± 0.08 log units for older adults. Mean stereoacuity (Titmus Fly SO-001 StereoTest) was 43.3 ± 10.3″ for young adults and 47.1 ± 14.2″ for older adults. All the measurements including main experiments were measured binocularly. The experimental protocols followed the tenets of the Declaration of Helsinki and were approved by the Internal Review Board (IRB) at the University of Alabama at Birmingham. Written informed consent was obtained from all participants prior to the experiment after explanation of the nature of the study.

### Stimulus and Apparatus

For reading speed and visual span tasks, the 26 Courier font letters of the English alphabet were used. For crowding zone task, ten Sloan letters and tumbling ‘E’s with different orientations (0°, 90°, 180°, 270°) were used as the target letter and flankers, respectively. For all tests, letters were black on a uniform gray background (159  *cd*/*m* ^2^) with a contrast of 99%. Letter size, defined as x-height, was 0.8°. All stimuli were generated and controlled using MATLAB (version 8.3) and Psychophysics Toolbox extensions^96, 97^ for Windows 7, running on a PC desktop computer (model: Dell Precision Tower 5810). Stimuli were presented on a liquid crystal display monitor (model: Asus VG278HE; refresh rate: 144 Hz; resolution: 1920 × 1080, subtending 60° × 34° visual angle at a viewing distance of 57 cm) with the mean luminance of the monitor at 159 *cd/m* ^2^. Luminance of the display monitor was made linear using an 8-bit look-up table in conjunction with photometric readings from a MINOLTA LS-110 Luminance Meter (Konica Minolta Inc., Japan).

### Measuring reading speed

Reading speed was measured with static text (Flashcard method^22, 74, 75^). The same method has been used in previous studies^22, 72^. All sentences were 56 characters in length and formatted into four lines of 14 characters. The mean difficulty of the sentences was below 3^rd^ grade level. Different reading measures likely tap into different aspects of the reading process. In this study, we focused on the role of vision in age-related decline in reading while minimizing high-level cognitive and linguistic influences. In that aspect, although the Flashcard reading is not natural, it was the best possible choice on the following grounds: (1) As the method encourages subjects to read as fast and accurately possible, it can maximize any limiting effect of front-end visual factors on reading speed; (2) Unlike silent reading, subjects are not allowed to skip words to simply read faster; (3) It is a proven method to examine the role of vision in reading speed for both normal and low vision^22, 72, 74–76^. For example, Flashcard reading speed was shown to be significantly correlated with developmental changes in the size of the visual span^22^.

The method of constant stimulus was used to present sentences at five exposure times, spanning a range of ~1.4 log unit. Participants were instructed to read the sentences aloud as quickly and accurately as possible. They were allowed to complete their verbal response at their own speed, not under time pressure. Five sentences were tested for each exposure time and the percent correct of word recognition was computed at each exposure time. Psychometric function, percent correct versus exposure time in seconds per sentence, was created by fitting data with cumulative Gaussian functions^98^. The threshold exposure time, defined as the exposure time yielding 80% of words read correctly, was then converted into the number of words read correctly per minute (wpm).

### Measuring crowding zone

**Figure 6:**
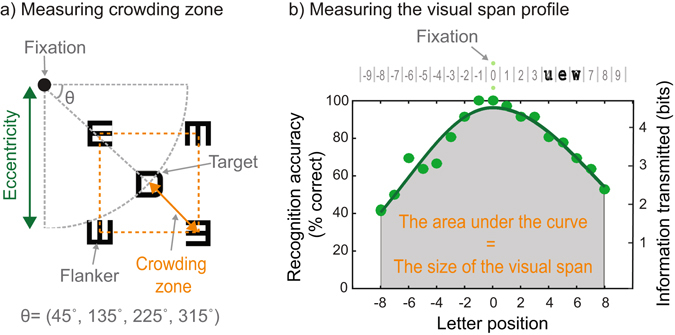
Task stimuli and procedure. (**a**) Measuring crowding zone. A target letter was located at one of three distances from the fixation (eccentricities = 2°, 4°, or 8°), and at one of four azimuth angles around the fixation (Ɵ = 45°, 135°, 225°, or 315°). In this example, the azimuth angle of the target ‘D’ is 315°. Four flankers (tumbling ‘E’s with different orientations) were located at four diagonal locations with the same distance from the target. Crowding zone represents the distance between the target and the flankers required to yield 79.4% letter-recognition accuracy. (**b**) Measuring the visual span profile. Top: an example of a trigram presented in a horizontal line. A trigram, a random string of three letters (e.g., ‘uew’), is centered at one of 19 positions in a horizontal line, left or right of fixation. Bottom: an example of visual span profile. A visual span profile consists of letter recognition accuracy as a function of letter position, and is fitted with a split Gaussian function. The right vertical scale shows a transformation from recognition accuracy to information transmitted in bits. The size of the visual span was defined as the area under the curve.

Crowding zone was assessed using a letter recognition task (See Fig. 6a). A target letter was located at one of three retinal eccentricities (2°, 4°, or 8°), and at one of four azimuth angles around the fixation (45°, 135°, 225°, or 315°). The location of the target was predetermined for each block and counterbalanced across blocks to minimize order effect. At the beginning of each block (11 trials), a small red dot was shown at the target location to cue participants for the target location. In each trial, a target letter with or without flankers (single-letter condition) was shown at the assigned location for 150 ms. Participants were instructed to keep fixating a central dot and to choose the target letter they recognized in a following letter panel. The spatial separation between the target and the flankers in each trial was determined by a 3-down 1-up staircase procedure^99^. Crowding zone was defined as the geometric mean of the last 6 reversals out of total 7 reversals in the staircase procedure for each location, representing the distance between the target and the flankers required to yield 79.4% letter-recognition accuracy.

### Measuring visual-span profiles

Visual-span profiles were measured using a trigram letter-recognition task. The procedure was similar to previous studies^22, 71^. Trigrams (random strings of three random letters) were centered at one of 19 positions in a horizontal line (see Fig. 6b). Each trigram was flashed for 200 ms followed by a post-mask (a string of letter ‘X’s). Participants were asked to fixate between two fixation points and to report the three letters from left to right. A letter was scored as being identified correctly only if its order within the trigram was also correct. Each trigram position was tested 12 times, in a random order. A visual span profile was fitted with a split Gaussian function based on the recognition accuracy at each letter slot^22, 71^. The size of the visual span was defined as the area under the profile, and was quantified in units of bits of information transmitted^100^.

Throughout the testing sessions, participants’ compliance with central fixation was continuously ensured either via a high-speed eyetracker or a webcam. Besides, the stimulus duration of 200 ms or less was too brief for saccadic eye movements.

### Data analysis

Statistical analyses were performed using IBM SPSS statistics (version 23) and Matlab R2014b (MathWorks Inc, U.S.). Quantile-Quantile plot was used to confirm the data were normally distributed. Two-tailed independent samples t-test was applied to compare the difference in reading speed, the size of the visual span and crowding zone between the two age groups. When the data was not normally distributed, Mann-Whitney U test was run instead of independent sample t-test. A repeated measures analysis of variance (ANOVA) was used to examine the main effect of age group or eccentricity on crowding zone and single-letter recognition accuracy, respectively. Person product-moment correlation was run to analyze the correlation between reading speed and each of the two crowding measures.

### Data availability

The datasets generated and analyzed during the current study are available from the corresponding author on reasonable request.
